# Supplementary material for: PLK1‐dependent phosphorylation restrains EBNA2 activity and lymphomagenesis in EBV‐infected mice
Source: EMBO Rep. 2021 Oct 4;22(12):e53007. doi: 10.15252/embr.202153007 (PMC8647151; doi:10.15252/embr.202153007)
Supplement: Supplementary file 1 — Expanded View Figures PDF [file EMBR-22-e53007-s002.pdf]

Expanded View Figures

Figure EV1. Annotated HCD MS/MS spectra of phosphopeptides.

A–E (A) LVQPHVPPLRPTAPTILSPLSQPR, (B) MHLPVLHVPDQSMHPLTHQSTPNDPDSPEPR, (C) DLDESWDYIFETTESPSSDER, (D) TTESPSSDEDYVEGPSKRPRPSIQ, and (E) DYVEGPSKRPRPSIQ, bearing 5 confidentially localized phosphorylation sites, S184, 258, 457, T465, and S479, respectively. The "ph" denotes phosphosites localized. The a-, b-, and y-ions are in pale blue, dark blue, and red, respectively. Ions with neutral losses are in orange, internal fragment ions in purple, ammonium ion in green, and side-chain loss in turquoise. The asterisk (*) denotes loss of H_3O_4P with a delta mass of 97.9768 from the phosphorylated fragment ion.

 © 2021 The Authors

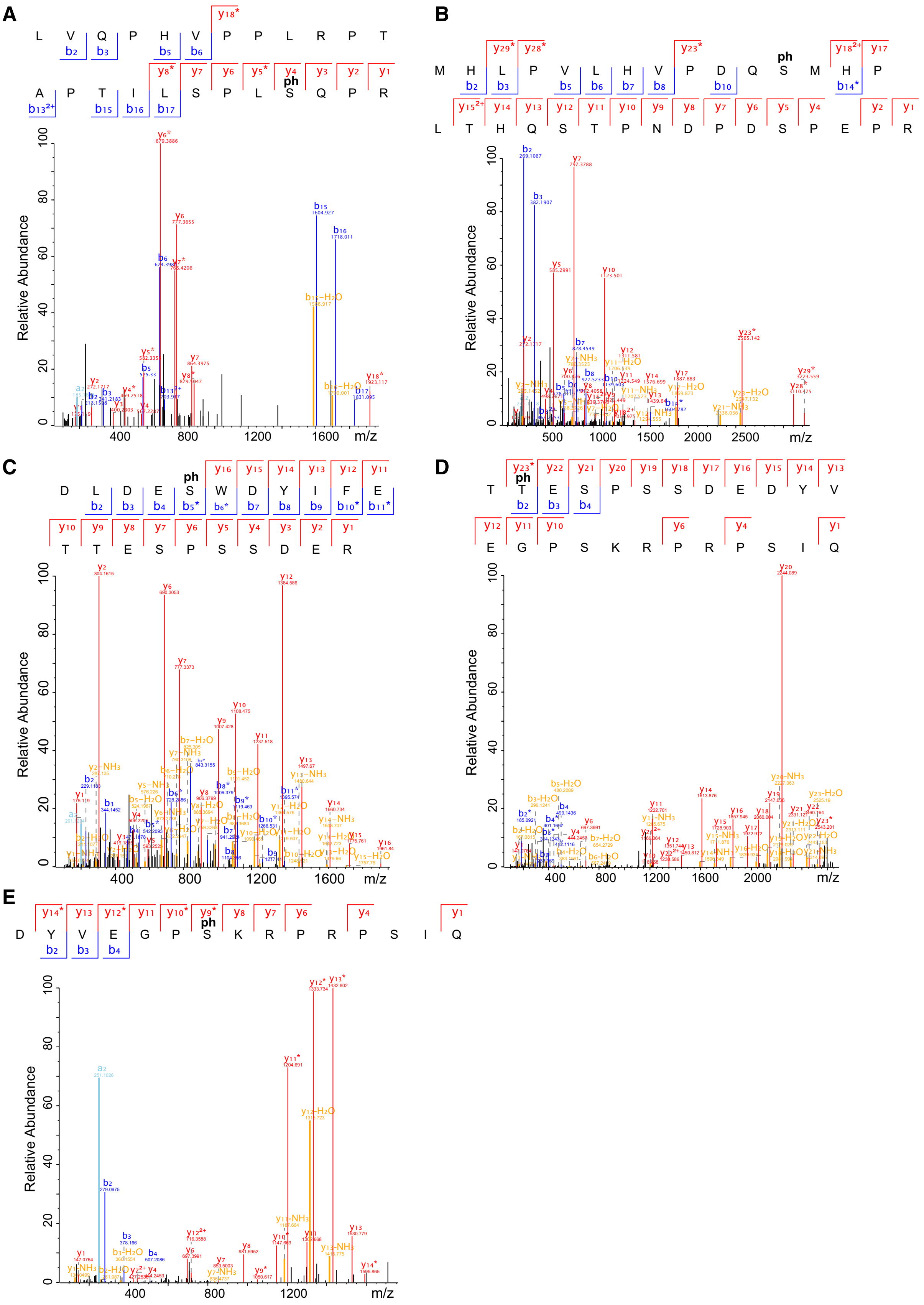

Figure EV1.

© 2021 The Authors

Figure EV2. Overview of sequence coverage and phosphorylation sites identified by mass spectrometry.

A, B (A) 6x His-tagged EBNA2 and (B) GST-EBNA2 453–474 phosphorylated by PLK1. Proteins were expressed in E. coli and extracted after SDS–PAGE separation, digested by trypsin (green bars) and V8 (blue bars) in parallel and submitted to LC-MS/MS (enlarged letters in red or green). The sign # denotes Arg (R) inserted to facilitate fragmentation. The asterisk (*) denotes the initial Met (M) of EBNA2. Proteins before phosphorylation by PLK1 were studied in parallel but no phosphorylation sites were identified. The sequence corresponds to UniProt ID: P12978.1.

 © 2021 The Authors

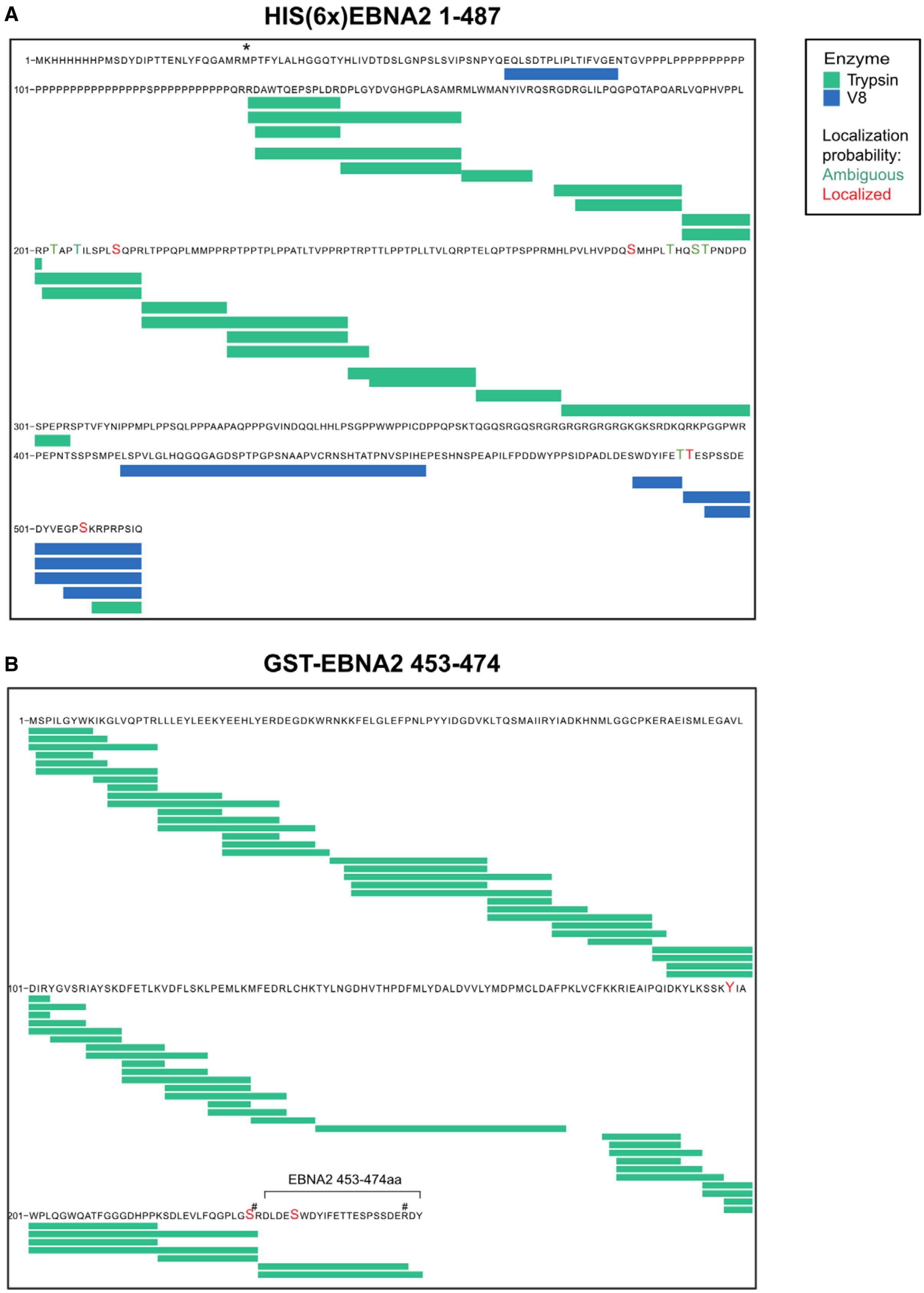
A
HIS(6x)EBNA2 1-487
B
GST-EBNA2 453-474
Enzyme
Trypsin
V8
Localization probability:
Ambiguous
Localized
EBNA2 453-474aa

Figure EV2.

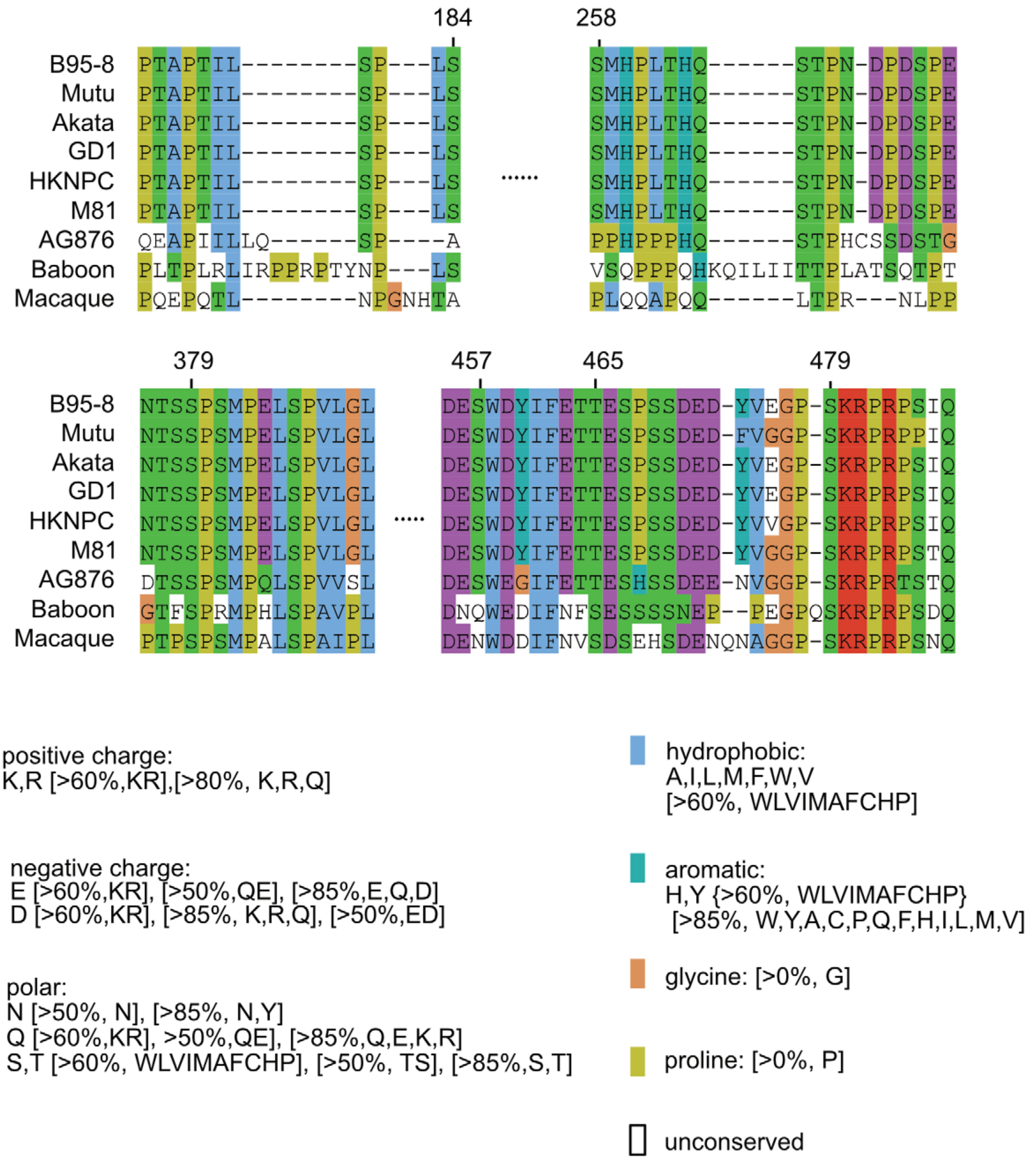

Figure EV3. Sequence alignment of EBNA2 expressed by type 1 and type 2 EBV or by baboon and macaque lymphocryptoviruses.

Multiple sequence alignment of EBNA2 type 1 (B95-8, Mutu, Akata, GD1, HKNPC, and M81), type 2 (AG876) and the EBNA2 paralogs of baboon and macaque lymphocryptoviruses generated by MUSCLE (Edgar, 2004). The numbering of amino acid residues is based on EBNA2 B95-8 (P12978.1). Each residue in the alignment is assigned a color if the amino acid profile of the alignment at that position meets some minimum criteria specific for the residue type. The color code (http://www.jalview.org/help/html/colourSchemes/clustal.html) below the alignment gives these criteria as clauses: [>X%,xx,y], where X is the threshold percentage presence for any of the xx (or y) residue types.

 © 2021 The Authors

Figure EV4. Construction of EBV BACmids carrying HA-tagged EBNA2 mutants impaired for PLK1 binding or PLK1 phosphorylation and functional tests in cell culture.

A Electrophoretic separation of the restriction digest of EBV Bac DNA: p6008 (precursor), pXZ135 (insertion of Kan/rpsL as a precursor for EBV HA-EBNA2), and pXZ143 (EBV HA-EBNA2=EBV wt). The arrows highlight distinct fragments that characterize the individual BACs in size upon Kan/rpsL insertion and deletion (6,005 bp → 7,353 bp → 6,035 bp). Molecular markers: λ DNA-Hind III digest (nonitalics) and λ DNA-BstE II digest (italics). (#) denotes the fragment derived from fragments denoted by asterisks (*).

B Sanger sequencing of pXZ143 to confirm the insertion of the HA-tag into EBNA2 in the backbone of p6008.

C, D Sanger sequencing of pXZ203 (C) and pXZ146 (D) to confirm the substitution of S379A and S457A/T465V, respectively.

E Gating strategy of cell trace violet-stained active B cells after EBV infection.

F Adenoid B cells were stained with cell trace violet before they were infected with EBV mutants as indicated and analyzed by flow cytometry.

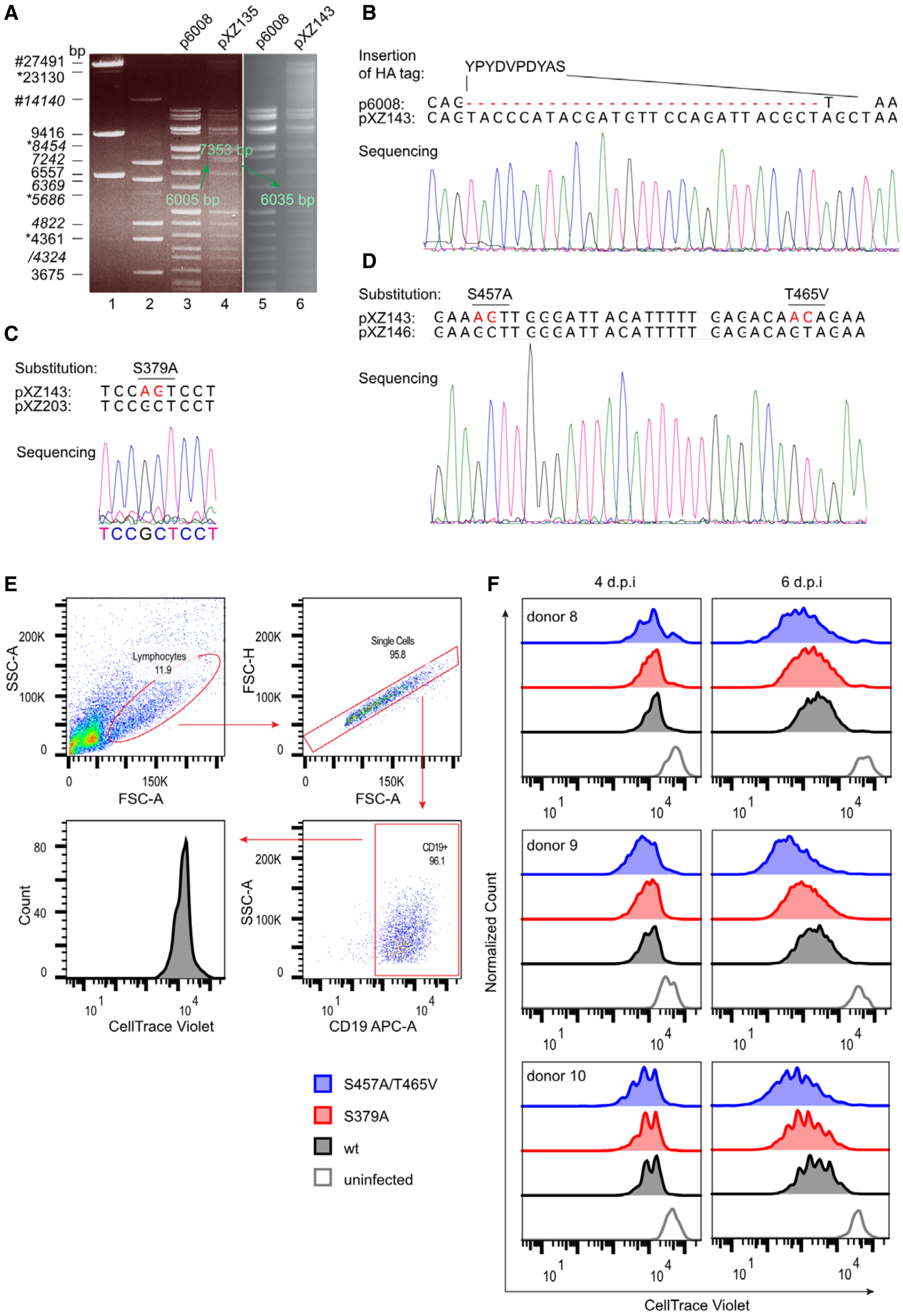

Figure EV4.

 © 2021 The Authors

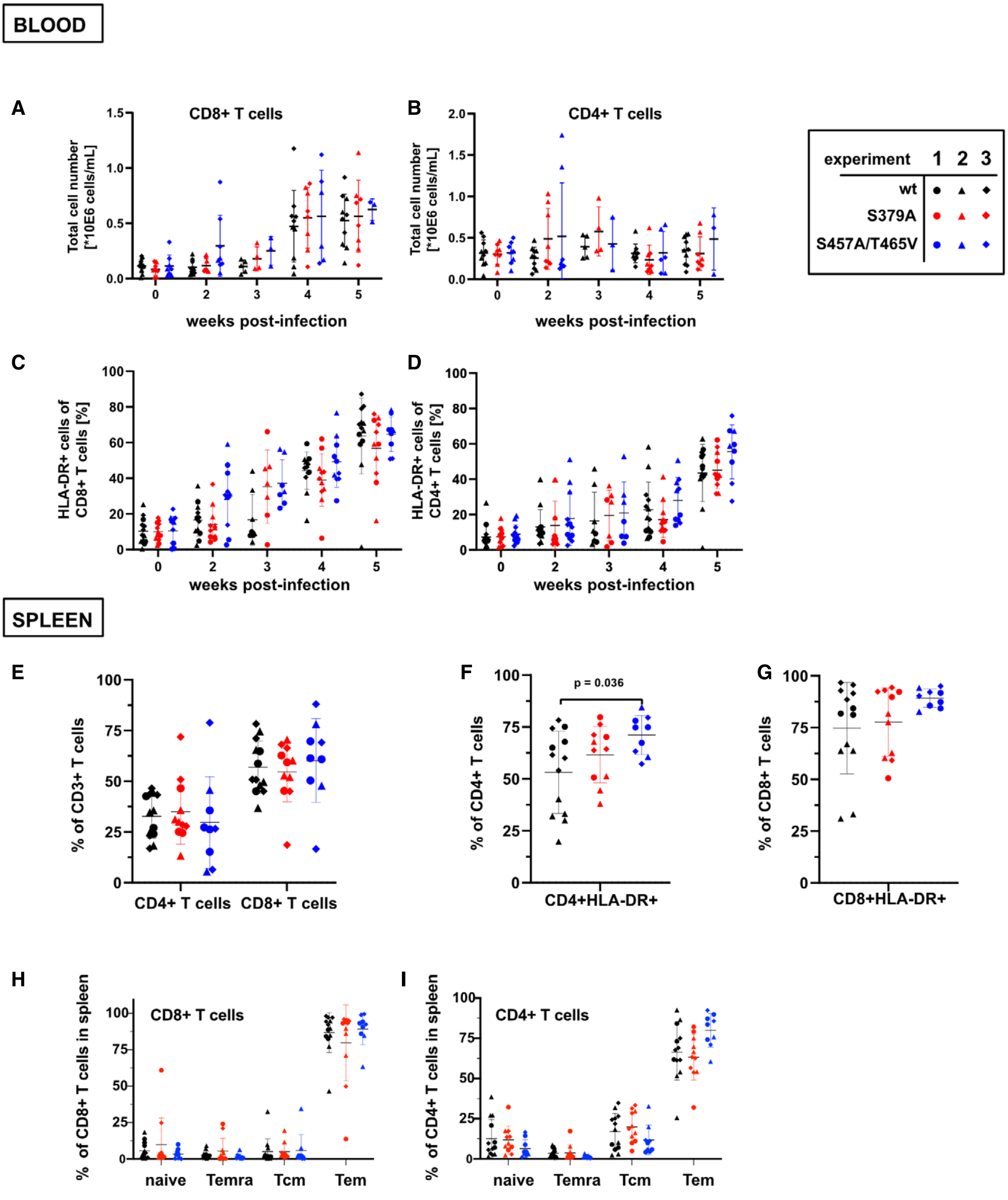
BLOOD
A
CD8+ T cells
Total cell number [*10E6 cells/mL]
weeks post-infection
B
CD4+ T cells
Total cell number [*10E6 cells/mL]
weeks post-infection
experiment 1 2 3
wt
S379A
S457A/T465V
C
HLA-DR+ cells of CD8+ T cells [%]
weeks post-infection
D
HLA-DR+ cells of CD4+ T cells [%]
weeks post-infection
SPLEEN
E
% of CD3+ T cells
CD4+ T cells CD8+ T cells
F
p = 0.036
% of CD4+ T cells
CD4+HLA-DR+
G
% of CD8+ T cells
CD8+HLA-DR+
H
CD8+ T cells
% of CD8+ T cells in spleen
naive Temra Tcm Tem
I
CD4+ T cells
% of CD4+ T cells in spleen
naive Temra Tcm Tem

Figure EV5.

Figure EV5. T-cell subpopulations of blood and spleen of infected mice.

A–D Flow-cytometric analyses of (A) $CD8^+$ or (B) $CD4^+$ T-cell percentages and (C) $CD8^+$ and (D) $CD4^+$ T-cell activation in the blood of mice infected with wt or mutant EBV over a period of 5 weeks. Error bars indicate mean ± SD. Number of biological replicates per group: EBNA2 WT EBV weeks 0, 2, 4, and 5: n = 10, week 3: n = 5; EBNA2 S379A weeks 0, 2, 4, and 5: n = 8, week 3: n = 4; EBNA2 S457A T465V weeks 0 and 2: n = 8, week 3: n = 3, week 4: n = 6, week 5: n = 3.

E–I (E) Analyses of $CD4^+$ and $CD8^+$ T-cell subpopulations and activation of (F) $CD4^+$ and (G) $CD8^+$ subpopulations in the spleen. Frequencies of (H) $CD8^+$ and (I) $CD4^+$ naïve, terminally differentiated (Temra), central memory (TCM), and effector memory (Tem) T-cell subpopulations. Error bars indicate mean ± SD. Number of biological replicates per group: EBNA2 WT: n = 13; EBNA2 S379A: n = 11; EBNA2 S457A T465V: n = 9.

Data information: The shape of data points indicates to which cohort (experiment 1, 2, or 3) the respective animal belongs while the color indicates the EBV EBNA2 mutant with which the animal was infected. Statistical significance was tested using the Mann–Whitney *U*-test with Holm–Sidak correction for multiple comparisons.

 © 2021 The Authors
