# Supplementary material for: PLK1‐dependent phosphorylation restrains EBNA2 activity and lymphomagenesis in EBV‐infected mice
Source: EMBO Rep. 2021 Oct 4;22(12):e53007. doi: 10.15252/embr.202153007 (PMC8647151; doi:10.15252/embr.202153007)
Supplement: Supplementary file 2 — Table EV1 [file EMBR-22-e53007-s003.docx]

| ensemble-ID | Peptide count^a^ | Peptides used for quantification^b^ | Confidence score^c^ | Anova (p) | Accumulation^d^ | Symbol | Replicate 1^e^ vector (empty) | Replicate 2^e^ vector (empty) | Replicate 3^e^ vector (empty) | Replicate 1^e^ EBNA2 wt | Replicate 2^e^ EBNA2 wt | Replicate 3^e^ EBNA2 wt | Description |
| --- | --- | --- | --- | --- | --- | --- | --- | --- | --- | --- | --- | --- | --- |
| ENSP00000248958 | 1 | 1 | 48 | 0.00 | 1652 | SDF2L1 | 27 | 0 | 0 | 4799 | 33098 | 6503 | stromal cell-derived factor 2-like 1 |
| ENSP00000247020 E | 2 | 2 | 54 | 0.01 | 762 | SDF2 | 26 | 0 | 0 | 2392 | 14742 | 2515 | stromal cell-derived factor 2 |
| ENSP00000342070 | 2 | 2 | 85 | 0.04 | 177 | CTSB | 56 | 0 | 36 | 11264 | 330 | 4616 | cathepsin B |
| ENSP00000380376 | 1 | 1 | 34 | 0.01 | 164 | PAXIP1 | 13 | 27 | 2 | 338 | 4697 | 1748 | PAX interacting (with transcription-activation domain) protein 1 |
| ENSP00000311766 | 13 | 1 | 336 | 0.04 | 125 | ATAD3B | 59 | 37 | 0 | 469 | 9408 | 2205 | ATPase family, AAA domain containing 3B |
| ENSP00000300093 | 7 | 7 | 172 | 0.01 | 77 | PLK1 | 1610 | 407 | 198 | 14583 | 122709 | 32353 | polo-like kinase 1 |
| ENSP00000265028 | 5 | 5 | 207 | 0.01 | 46 | DNAJB11 | 1645 | 2063 | 2421 | 20887 | 211659 | 49942 | DnaJ (Hsp40) homolog, subfamily B, member 11 |
| P12978.1 | 5 | 5 | 162 | 0.01 | 26 | EBNA2 | 5841 | 3949 | 3629 | 41868 | 251544 | 59586 | Epstein-Barr nuclear antigen 2 |
| ENSP00000305815 | 1 | 1 | 26 | 0.01 | 26 | CBF1/RBPJ | 410 | 121 | 93 | 1602 | 10431 | 4182 | recombination signal binding protein for immunoglobulin kappa J region |
| ENSP00000261893 | 7 | 7 | 210 | 0.01 | 17 | LACTB | 5688 | 3130 | 2773 | 21048 | 129818 | 47963 | lactamase, beta |
| ENSP00000368030 | 18 | 6 | 544 | 0.03 | 11 | ATAD3A | 13872 | 9774 | 4851 | 41200 | 213392 | 45267 | ATPase family, AAA domain containing 3A |
| ENSP00000446596 | 3 | 2 | 105 | 0.05 | 10 | DYNLL1 | 13993 | 9084 | 6329 | 25470 | 217534 | 46037 | dynein, light chain, LC8-type 1 |
| ENSP00000277900 | 5 | 4 | 100 | 0.05 | 7 | ADD3 | 6551 | 765 | 819 | 12200 | 35354 | 9851 | adducin 3 (gamma) |
| ENSP00000306223 | 2 | 2 | 32 | 0.03 | 4 | SNRPN | 508 | 154 | 254 | 1595 | 1306 | 639 | small nuclear ribonucleoprotein polypeptide N |
| ENSP00000280326 | 2 | 2 | 47 | 0.05 | 2 | CCT5 | 950 | 950 | 1819 | 1867 | 3216 | 4077 | chaperonin containing TCP1, subunit 5 (epsilon) |
| ENSP00000295688 | 2 | 2 | 45 | 0.03 | 2 | CCT3 | 1269 | 1133 | 1066 | 1579 | 1979 | 2715 | chaperonin containing TCP1, subunit 3 (gamma) |
| ENSP00000261182 | 1 | 1 | 35 | 0.01 | 2 | NAP1L1 | 1081 | 843 | 707 | 1562 | 1580 | 1540 | nucleosome assembly protein 1-like 1 |
| ENSP00000272163 | 1 | 1 | 29 | 0.01 | 2 | LBR | 1550 | 1597 | 1653 | 2133 | 3232 | 2798 | lamin B receptor |
| ENSP00000238081 | 4 | 3 | 123 | 0.00 | 2 | YWHAQ | 5744 | 5869 | 7103 | 9673 | 10151 | 10026 | tyrosine 3-monooxygenase/tryptophan 5-monooxygenase activation protein, theta polypeptide |

**Table EV1: 19 candidate EBNA2 associated proteins identified by label free mass spectrometry**

**Footnotes**

^a^ Number of peptides detected during mass spectrometric analysis.

^b^ only peptides specific for the protein were used for quantification.

^c^ The confidence score reflects the combined scores of all observed mass spectra that can be matched to amino acid sequences within that protein. A higher score indicates a more confident match.

^d^ This value reflects the accumulation of proteins found after immunoprecipitation using EBNA2 wt as a bait compared to the proteins found using no bait (empty vector). The value was calculated by dividing the mean values of the normalized abundances (see footnote ^e^).

^e^ Normalized abundances derived from mass spectrometric analysis.
