# Supplementary figures and images for: PLK1‐dependent phosphorylation restrains EBNA2 activity and lymphomagenesis in EBV‐infected mice
Source: EMBO Rep. 2021 Oct 4;22(12):e53007. doi: 10.15252/embr.202153007 (PMC8647151; doi:10.15252/embr.202153007)

Fig.1B

Fig. 1A

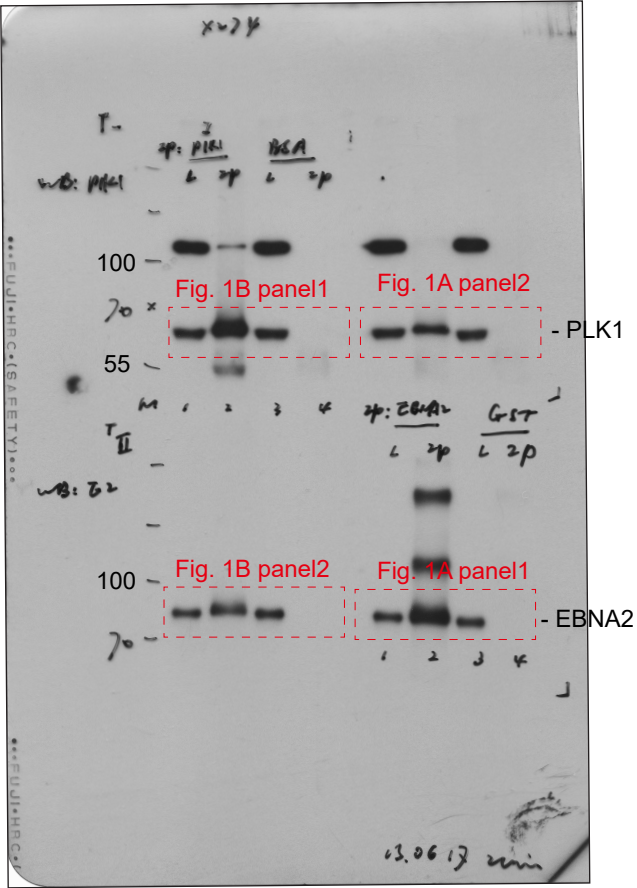

Supplement: Supplementary file 4 — Source Data for Figure 1 [file EMBR-22-e53007-s007.pdf]

Fig. 2B

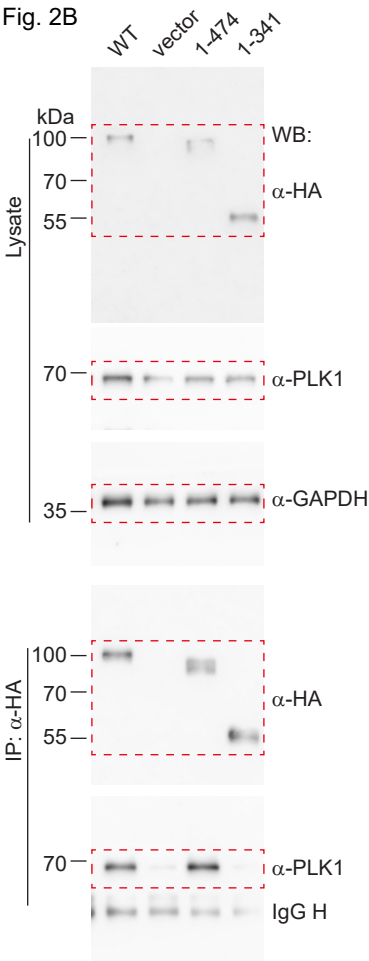

Fig. 2E

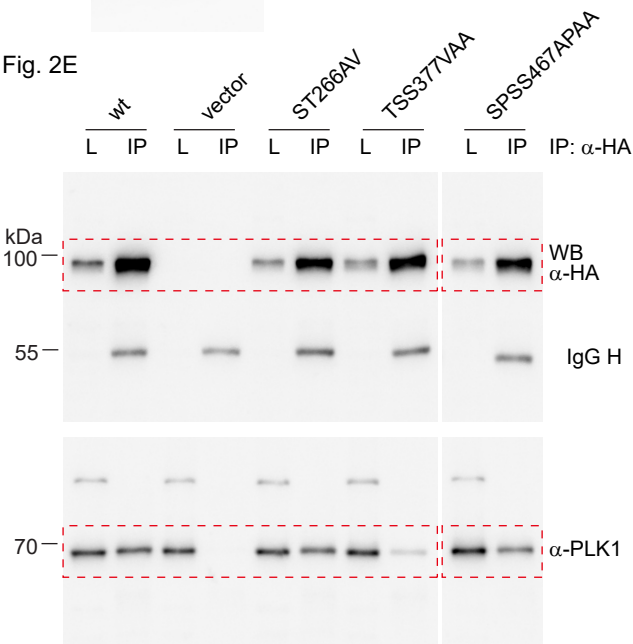

Fig. 2G

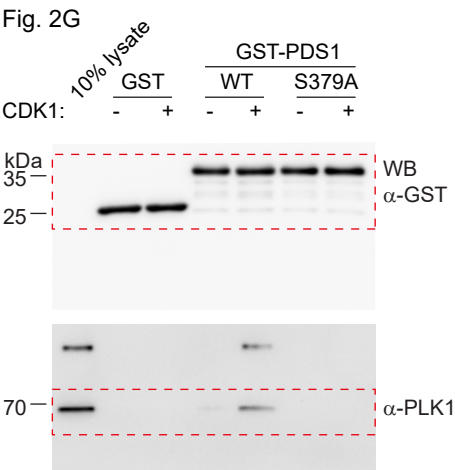

Fig. 2C

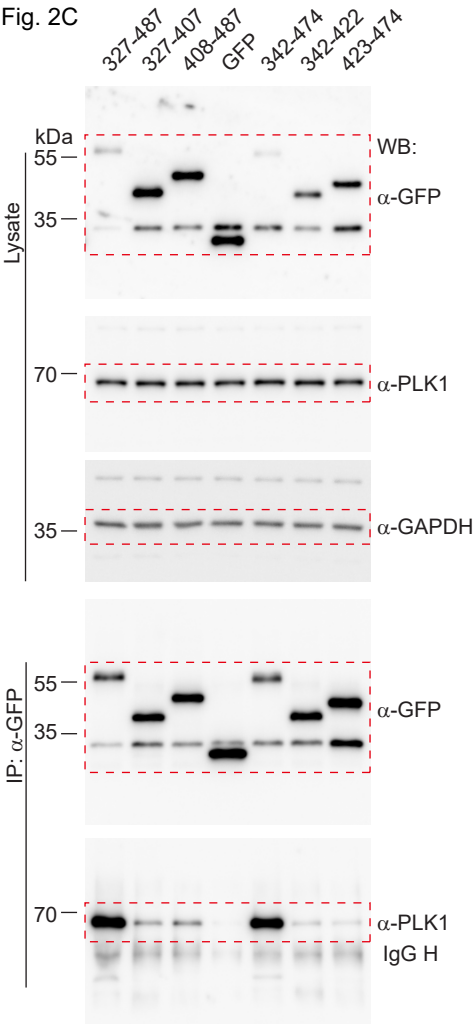

Fig. 2H

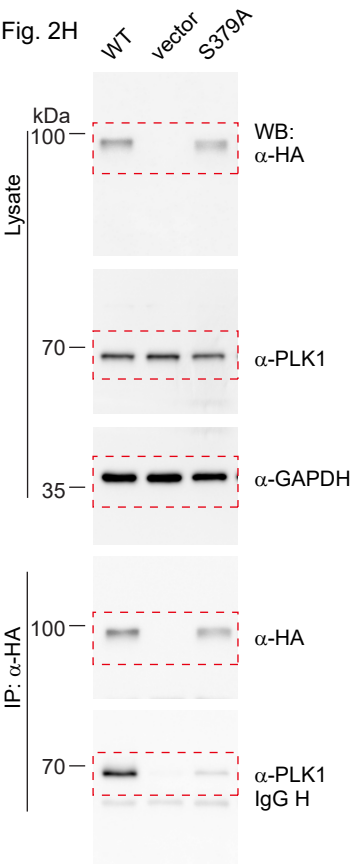

Supplement: Supplementary file 5 — Source Data for Figure 2 [file EMBR-22-e53007-s004.pdf]

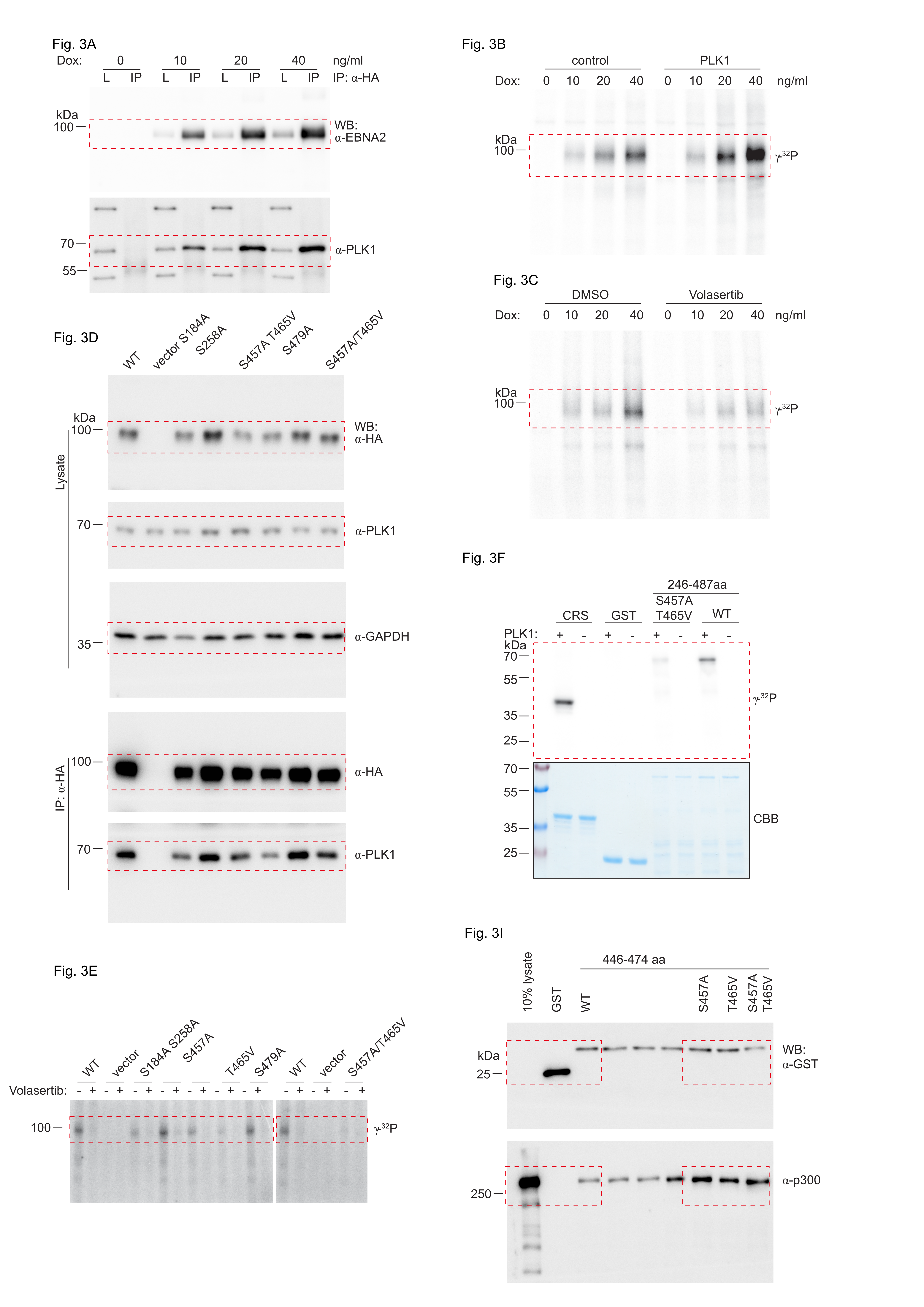

Supplement: Supplementary file 6 — Source Data for Figure 3 [file EMBR-22-e53007-s001.tif]

Fig. 4B

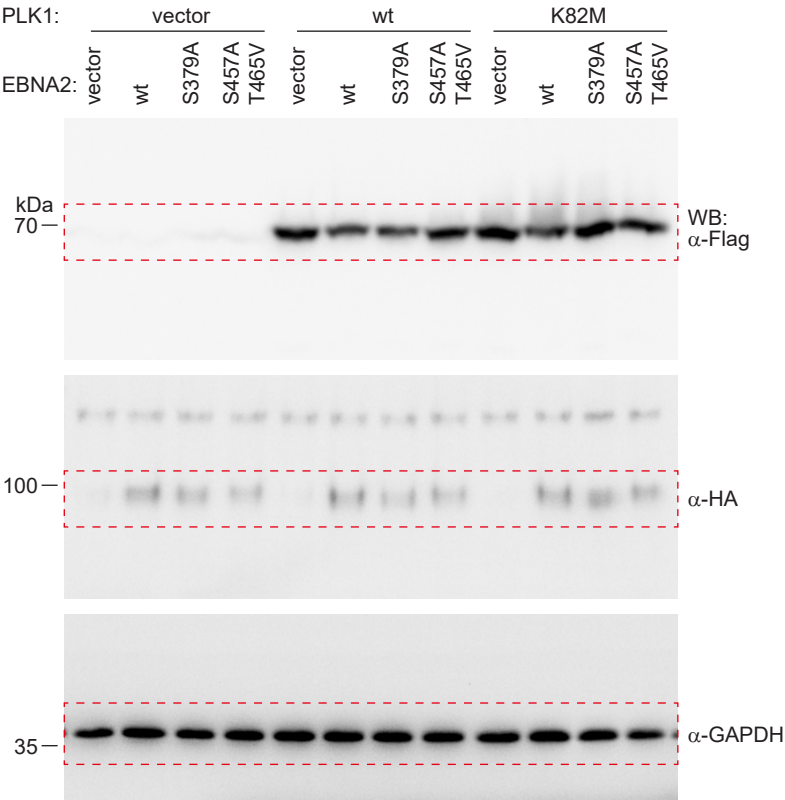

Supplement: Supplementary file 7 — Source Data for Figure 4 [file EMBR-22-e53007-s005.pdf]

Fig. 5C

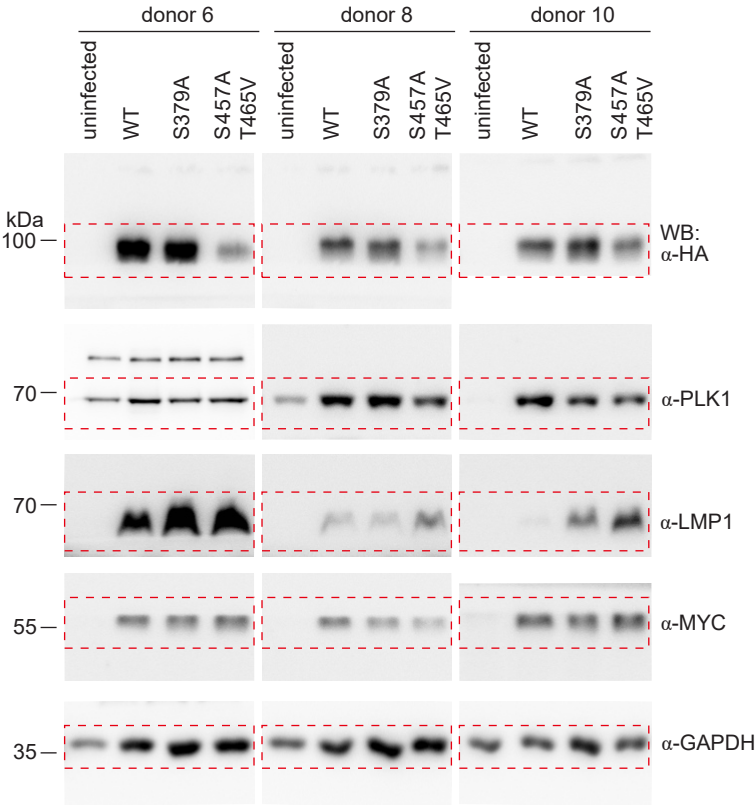

Supplement: Supplementary file 8 — Source Data for Figure 5 [file EMBR-22-e53007-s008.pdf]
