# Supplementary material for: PLK1‐dependent phosphorylation restrains EBNA2 activity and lymphomagenesis in EBV‐infected mice
Source: EMBO Rep. 2021 Oct 4;22(12):e53007. doi: 10.15252/embr.202153007 (PMC8647151; doi:10.15252/embr.202153007)
Supplement: Supplementary file 9 [file EMBR-22-e53007-s006.pdf]

PLK1-dependent phosphorylation restrains EBNA2 activity and lymphomagenesis in EBV-infected mice

Xiang Zhang, Patrick Schuhmachers, Andre Mourao, Piero Giansanti, Anita Murer, Sybille Thumann, Cornelia Kuklik-Roos, Sophie Beer, Stefanie Hauck, Wolfgang Hammerschmidt, Ralf Küppers, Bernhard Küster, Monika Raab, Klaus Strebhardt, Michael Sattler, Christian Münz, and Bettina Kempkes
DOI: 10.15252/embr.202153007

Corresponding author(s): Bettina Kempkes (kempkes@helmholtz-muenchen.de) , Michael Sattler (sattler@helmholtz-muenchen.de), Xiang Zhang (xiang.zhang.1118@gmail.com)

Review Timeline:

Submission Date:	6th Apr 21
Editorial Decision:	28th Apr 21
Revision Received:	11th Jun 21
Editorial Decision:	9th Jul 21
Revision Received:	17th Aug 21
Accepted:	10th Sep 21

Editor: Achim Breiling/Martina Rembold

Transaction Report:

(Note: With the exception of the correction of typographical or spelling errors that could be a source of ambiguity, letters and reports are not edited. Depending on transfer agreements, referee reports obtained elsewhere may or may not be included in this compilation. Referee reports are anonymous unless the Referee chooses to sign their reports.)

1st Editorial Decision **28th Apr 2021**

Dear Dr. Kempkes,

Thank you for the submission of your research manuscript to EMBO reports. We have now received the reports from the referees that were asked to evaluate your study, which can be found at the end of this email.

As you will see, the referees think that these findings are of interest. However, they have several comments, concerns and suggestions, indicating that a major revision of the manuscript is necessary to allow publication of the study in EMBO reports. As the reports are below, and I think all their points need to be addressed, I will not detail them here.

Given the constructive referee comments, we would like to invite you to revise your manuscript with the understanding that all referee concerns must be addressed in the revised manuscript or in the detailed point-by-point response. Acceptance of your manuscript will depend on a positive outcome of a second round of review. It is EMBO reports policy to allow a single round of revision only and acceptance of the manuscript will therefore depend on the completeness of your responses included in the next, final version of the manuscript.

Revised manuscripts should be submitted within three months of a request for revision. We are aware that many laboratories cannot function at full efficiency during the current COVID-19/SARS-CoV-2 pandemic and we have therefore extended our 'scooping protection policy' to cover the period required for full revision. Please contact me to discuss the revision should you need additional time, and also if you see a paper with related content published elsewhere.

When submitting your revised manuscript, please also carefully review the instructions that follow below.

PLEASE NOTE THAT upon resubmission revised manuscripts are subjected to an initial quality control prior to exposition to re-review. Upon failure in the initial quality control, the manuscripts are sent back to the authors, which may lead to delays. Frequent reasons for such a failure are the lack of the data availability section (please see below) and the presence of statistics based on n=2 (the authors are then asked to present scatter plots or provide more data points).

When submitting your revised manuscript, we will require:

1) a .docx formatted version of the final manuscript text (including legends for main figures, EV figures and tables), but without the figures included. Please make sure that changes are highlighted to be clearly visible. Figure legends should be compiled at the end of the manuscript text.

2) individual production quality figure files as .eps, .tif, .jpg (one file per figure), of main figures and EV figures. Please upload these as separate, individual files upon re-submission.

The Expanded View format, which will be displayed in the main HTML of the paper in a collapsible format, has replaced the Supplementary information. You can submit up to 5 images as Expanded View. Please follow the nomenclature Figure EV1, Figure EV2 etc. The figure legend for these should be included in the main manuscript document file in a section called Expanded View Figure Legends after the main Figure Legends section. Additional Supplementary material should be supplied as a single pdf file labeled Appendix. The Appendix should have page numbers and needs

to include a table of content on the first page (with page numbers) and legends for all content. Please follow the nomenclature Appendix Figure Sx, Appendix Table Sx etc. throughout the text, and also label the figures and tables according to this nomenclature.

For more details, please refer to our guide to authors:
http://www.embopress.org/page/journal/14693178/authorguide#manuscriptpreparation

See also our guide for figure preparation:
http://wol-prod-cdn.literatumonline.com/pb-assets/embo-site/EMBOPress_Figure_Guidelines_061115-1561436025777.pdf

3) a .docx formatted letter INCLUDING the reviewers' reports and your detailed point-by-point responses to their comments. As part of the EMBO Press transparent editorial process, the point-by-point response is part of the Review Process File (RPF), which will be published alongside your paper.

4) a complete author checklist, which you can download from our author guidelines (https://www.embopress.org/page/journal/14693178/authorguide). Please insert page numbers in the checklist to indicate where the requested information can be found in the manuscript. The completed author checklist will also be part of the RPF.

Please also follow our guidelines for the use of living organisms, and the respective reporting guidelines: http://www.embopress.org/page/journal/14693178/authorguide#livingorganisms

5) that primary datasets produced in this study (e.g. RNA-seq, ChIP-seq and array data) are deposited in an appropriate public database. If no primary datasets have been deposited, please also state this in the respective section (e.g. 'No primary datasets have been generated and deposited'), see below.

See also: http://embor.embopress.org/authorguide#datadeposition

Please remember to provide a reviewer password if the datasets are not yet public.

The accession numbers and database should be listed in a formal "Data Availability " section (placed after Materials & Methods) that follows the model below. This is now mandatory (like the COI statement). Please note that the Data Availability Section is restricted to new primary data that are part of this study.

Data availability

The datasets produced in this study are available in the following databases:

- RNA-Seq data: Gene Expression Omnibus GSE46843 (https://www.ncbi.nlm.nih.gov/geo/query/acc.cgi?acc=GSE46843)
- [data type]: [name of the resource] [accession number/identifier/doi] ([URL or identifiers.org/DATABASE:ACCESSION])

*** Note - All links should resolve to a page where the data can be accessed. ***

Moreover, I have these editorial requests:

6) We strongly encourage the publication of original source data with the aim of making primary data more accessible and transparent to the reader. The source data will be published in a separate source data file online along with the accepted manuscript and will be linked to the relevant figure. If you would like to use this opportunity, please submit the source data (for example scans of entire gels or blots, data points of graphs in an excel sheet, additional images, etc.) of your key experiments together with the revised manuscript. If you want to provide source data, please include size markers for scans of entire gels, label the scans with figure and panel number, and send one PDF file per figure.

7) Our journal encourages inclusion of *data citations in the reference list* to directly cite datasets that were re-used and obtained from public databases. Data citations in the article text are distinct from normal bibliographical citations and should directly link to the database records from which the data can be accessed. In the main text, data citations are formatted as follows: "Data ref: Smith et al, 2001" or "Data ref: NCBI Sequence Read Archive PRJNA342805, 2017". In the Reference list, data citations must be labeled with "[DATASET]". A data reference must provide the database name, accession number/identifiers and a resolvable link to the landing page from which the data can be accessed at the end of the reference. Further instructions are available at:
http://www.embopress.org/page/journal/14693178/authorguide#referencesformat

8) Regarding data quantification and statistics, can you please specify, where applicable, the number "n" for how many independent experiments (biological replicates) were performed, the bars and error bars (e.g. SEM, SD) and the test used to calculate p-values in the respective figure legends. Please provide statistical testing where applicable, and also add a paragraph detailing this to the methods section. See:
http://www.embopress.org/page/journal/14693178/authorguide#statisticalanalysis

9) Please also note our new reference format:
http://www.embopress.org/page/journal/14693178/authorguide#referencesformat

Finally, please note that all corresponding authors are required to supply an ORCID ID for their name upon submission of a revised manuscript. Please find instructions on how to link the ORCID ID to the account in our manuscript tracking system in our Author guidelines:
http://www.embopress.org/page/journal/14693178/authorguide#authorshipguidelines

I look forward to seeing a revised version of your manuscript when it is ready. Please let me know if you have questions or comments regarding the revision.

Yours sincerely,

Achim Breiling
Editor
EMBO Reports

Referee #1:

The manuscript by Xiang and colleagues described studies where cellular PLK1 binds to EBNA2, phosphorylates its transactivation domain, and inhibits its biological activity to attenuate risk of tumor incidence. The investigators conducted clear and straightforward experiments to show

PLK1-dependent EBNA2 phosphorylation in B cell background.

However, the findings in Fig 5 and Fig 6 are not sufficient to demonstrate that the biological functions of EBNA2 mutants are mediated by PLK1. These experiments only showed that EBNA2 mutants (S379A and S457A/T465V) promote EBV-related tumorigenesis in vivo but may not be related to PLK1. This is a critical gap in this study.

Additionally, specific comments are listed below for the authors' consideration.

Page 6, line 189. The authors indicated PLK1 might negatively regulate EBNA2 activity in the luciferase reporter assay, but their results only provide supporting evidence that EBNA2 mutants carring either the S457A, T465V mutation, or both mutations showed enhanced transactivation activity. It is unknown whether the upregulation is mediated with PLK1 or not, so this conclusion is somehow overstated. Also, the figure (Fig 2H) is cited incorrectly here.

Fig 4 is not properly cited in the text.

The authors concluded that PLK1 acts as a tumor suppressor in EBV-driven pathogenesis (Page 3, line 108-109; Page 12, line 397-398). I am confused as to why they raise the possibility of treating the related tumors with PLK1 inhibitors (Page 3, line 101-102; Page 12, line 398-399). How do they explain these inconsistencies?

Referee #2:

This is a nice complete study identifying a new way that host cells can restrict the activity of the key Epstein-Barr virus transcription factor EBNA2. EBNA2 is an essential driver of the B cell transformation that enables EBV persistence in B cells and can ultimately lead to tumour development. Increasing evidence indicates that host cells use multiple mechanisms to suppress EBNA2 activity and that sequence variation in EBNA2 can affect its transactivation activity. This manuscript describes the identification of a new EBNA2 binding partner by mass spectrometry, the mitotic kinase PLK1, the mapping of its docking site (primed by phosphorylation by CDK1) and its phosphotarget sites in EBNA2. The authors demonstrate that PLK1 phosphorylation impairs EBNA2 transactivation activity in reporter assays and that recombinant viruses with mutations in PLK1 docking or phosphorylation sites drive the increased proliferation of infected B cells. These mutant viruses also establish B cell infections in humanized mice more rapidly and show increased tumour promoting properties linking the biochemical characterisation to an in vivo phenotype. Experiments are well executed and controlled, and conclusions are sound.

Suggestions for improvements are relatively minor:

1. Figure 3. S184A phosphorylation does seem to be reduced, contrary to the text, although transactivation assays rule out a functional role. Some additional phosphorylation sites may be mapped with lower confidence scores, rather than ambiguously.
2. It would be good to identify LMP1 and MYC as known EBNA2 target genes for the non EBV expert. The difference in impact on LMP1 activation by the mutants compared to MYC could be discussed. Could this relate to differences in dependency on p300 for activation? EBNA2 from type 2 EBV strains shows reduced activation of a subset of target genes including LMP1. Have the authors looked at gene activation profiles more widely? Can they draw any parallels?

3. I am not sure that I agree that 'EBNA2 activity needs to be controlled by PLK1'. Perhaps this is better described as a mechanism that is mutually beneficial to host and virus- reducing transformation and allowing establishment of latency.
4. The discussion section is quite repetitive of the results, often restating them. Could this section be used to expand on context and further hypotheses more? For example, could the balance of PLK1 phosphorylation and dephosphorylation be important in controlling transcription in interphase. Are these phosphorylation sites conserved across EBV strains?
5. Clarity of writing and use of English could be improved in places and there are incomplete/incorrect Figure citations (e.g. p4). Figure 1 legend and details do not state the identity of the cell lines used. The order of Figure 1 panels is not logical. The text on p8 implies that T cells are infected with mutant viruses.

Referee #3:

This manuscript provides new and exciting data on how EBNA2 is regulated and promotes oncogenesis. The study is initiated with a simple IP/MS experiment of tagged EBNA2 in DG75 cells. This brings down known interactors among 19 proteins and includes PLK1 as a novel interactor. The interaction is validated by western blotting and mutagenesis experiments suggests the PLK1 binding region of EBNA2 is in the C-terminus. Alignment of EBNA2 peptides with known PLK1 substrates identifies three potential sites where a CDK1 priming phosphorylation may enable PLK1 binding. Mutagenesis narrows this down to S379 and convincing evidence is provided for in vitro kinase (CDK1) activity and specific PLK1 binding of the phospho peptide as well as IP of EBNA2/PLK1 in cells. Next, EBNA2 is found to increase PLK1 activity and mutation of two key residues (S457 and T465) is found to ablate EBNA2 phosphorylation by PLK1. Interestingly, mutation of these residues increases EBNA2 transactivation potential on a CBF1-multimerized promoter. Furthermore, this mutant has increased association with the co-activator, p300 supporting the idea that PLK1 phosphorylation suppresses EBNA2 transcriptional activity, possibly through restricting p300 binding.

WT PLK1, but not a kinase dead mutant, suppressed EBNA2 activity when over-expressed, but this was lost on the PLK1 binding mutant, which robustly transactivated. Interestingly, the CDK1 priming mutant (S379A) was inhibited by PLK1 suggesting that the priming phosphorylation isn't required (at least in over-expression conditions) for PLK1 inhibition of EBNA2 activity.

Mutant EBV genomes were generated with S379A or S457A/T465V and interestingly these mutants both promoted outgrowth of latently infected B cells more so than wt virus. Carrying this phenotype over to huNSG mice, the authors find that the double mutant (which is unable to be suppressed by PLK1) kills mice, while the S379A and wt do not in this model of infection following CD34+ cell-mediated immune reconstitution. The difference in tumor burden is subtle, but notable where there is a higher proportion of macroscopically visible tumors in the double mutant mice (and perhaps single mutant). However, EBV viral loads in the spleen and blood are largely similar, though the mutant infections trend higher in the spleen and display a significant boost at 3 weeks post infection (the double mutant). Overall, these data paint an interesting molecular picture of EBNA2 regulation that follows on some earlier work by the Pagano lab indicating that EBNA2 transcriptional activity was suppressed by mitotic kinases.

Specific comments:
1. It would be interesting to know what the conservation of these aa residues are among EBV

strains (eg Farrell data set), type 1/2, and even back through NHPs.
2. It appears that the double mutant may be expressed lower in LCLs, could there be an impact on turnover (which negatively correlates with transcriptional activity?)
3. A transformation assay may provide better resolution on long-term impact of the mutants versus the short term proliferation assay.
4. Given the modest phenotype on tumorigenesis, perhaps the title should be adjusted to indicate that PLK1 restrains EBNA2 activity.

Referee #1:

The manuscript by Xiang and colleagues described studies where cellular PLK1 binds to EBNA2, phosphorylates its transactivation domain, and inhibits its biological activity to attenuate risk of tumor incidence. The investigators conducted clear and straightforward experiments to show PLK1-dependent EBNA2 phosphorylation in B cell background.
However, the findings in Fig 5 and Fig 6 are not sufficient to demonstrate that the biological functions of EBNA2 mutants are mediated by PLK1. These experiments only showed that EBNA2 mutants (S379A and S457A/T465V) promote EBV-related tumorigenesis in vivo but may not be related to PLK1. This is a critical gap in this study.

We need to say that Figure 4, not Figure 5/6 as suggested by referee 1, demonstrates that PLK1 impairs EBNA2 activity and thus provides the causal link between PLK1 and EBNA2 activity, using the EBNA2 mutants as discussed below. Figures 5 and 6 demonstrate the in vivo consequences of this specific interaction.

The results shown in Figure 4 demonstrate that PLK1 severely impairs EBNA2 wt functions. The negative control, the kinase dead PLK1, is not able to do so, indicating that kinase activity is required to attenuate EBNA2 activity. The binding mutant S379A is weakly affected by PLK1 co-expression while the phosphorylation mutant (S457A/T465V) is fully resistant to PLK1 inhibition. These results indicate that the residual binding of S379A to PLK1 (Figure 2H, last lane) still allows some weak inhibition. Our results firmly link EBNA2 phosphorylation by PLK1 to its biological activity in vitro and the relevance of these findings is confirmed by results we obtained in the animal model as shown in Figures 5 and 6. In vitro and in vivo, both mutants are gain of function mutants and the double mutant had the stronger phenotype.

The respective paragraph has been modified (line 208-219, page 6-7):

“To directly study if PLK1 inhibits EBNA2 functions, PLK1 and EBNA2 were co-expressed and luciferase reporter assays were performed (Figure 4A). In these transient transfection experiments, all EBNA2 mutants and all PLK1 mutants were expressed well (Figure 4B). EBNA2 wt activity was significantly reduced by co-expression of PLK1 but not affected by the kinase dead K82M PLK1 mutant. The activity of the docking site mutant S379A was weakly impaired by co-expression of PLK1 suggesting that residual binding activity that is evident in Figure 2H (last lane), might still recruit PLK1 activity. Importantly, the transactivation capacity of the phosphorylation mutant S457A/T465V was not affected by PLK1 co-expression demonstrating that EBNA2 S457A/T465V is resistant to PLK1 treatment. Since the kinase-dead PLK1 mutant did not impair the transactivation capacity of any EBNA2 protein, we conclude that PLK1 binding is necessary but not sufficient to inhibit EBNA2 and the phosphorylation of EBNA2 by PLK1 is required to inhibit EBNA2.“

In summary, for our in vivo studies (Figures 5 and 6), we have chosen two distinct mutants which we had extensively characterized in biochemical and functional in vitro cell culture experiments before. Both are impaired for two different distinct interactions with EBNA2 and thus provide two independent lines of evidence that support our conclusion.

Until to date, the interaction of EBNA2 with PLK1 was unknown and thus latent EBV was not considered to be a potential risk factor in clinical PLK1 inhibitor studies. To directly test if inhibition of PLK1 causes such a risk, pre-clinical animal models will need to be established for follow up studies to test PLK1 inhibition in humanized mice infected with EBV and EBV mutants.

These further very labor intensive and costly comprehensive studies were beyond the scope of our project but we agree they would be interesting to perform in the future.

Additionally, specific comments are listed below for the authors' consideration.

Page 6, line 189. The authors indicated PLK1 might negatively regulate EBNA2 activity in the luciferase reporter assay, but their results only provide supporting evidence that EBNA2 mutants carring either the S457A, T465V mutation, or both mutations showed enhanced transactivation activity. It is unknown whether the upregulation is mediated with PLK1 or not, so this conclusion is somehow overstated. Also, the figure (Fig 2H) is cited incorrectly here.

Fig 4 is not properly cited in the text.

This comment of referee #1 indicates that two mistakes we made might have caused some misunderstanding. Figure 2 H should have been named Figure 3H. Also, we did not refer to Figure 4 in the main text. We apologize for our mistakes and have corrected both in the revised manuscript.

We agree with referee 1 that the experiments described in Figure 3 are not sufficient to state that PLK1 inhibits EBNA2. As stated above the results shown in Figure 4 addressed this specific question.

The authors concluded that PLK1 acts as a tumor suppressor in EBV-driven pathogenesis (Page 3, line 108-109; Page 12, line 397-398). I am confused as to why they raise the possibility of treating the related tumors with PLK1 inhibitors (Page 3, line 101-102; Page 12, line 398-399). How do they explain these inconsistencies?

Unfortunately, our text on page 3 and 12 could be misunderstood. We never wanted to suggest the use of PLK1 inhibitors in EBV-related diseases. We rather suggest to carefully re-consider the present and potential indications for PLK1 inhibitors. As discussed in the text, PLK1 may act as oncogene or tumor suppressor. Whether it exerts either of the two functions depends on the cellular environment, defined by tissue origin, genetic lesions and potential additional factors that still need to be defined. Our study makes an important contribution to this specific bi-functional nature of PLK1. We demonstrate that in EBV transformed B cells that express EBNA2, PLK1 can act as a tumor suppressor.

Please find the modified text of the discussion section in lines 413-416.

Whether it exerts oncogenic or tumor suppressor functions depends on the cellular environment, defined by tissue origin, genetic lesions and potential additional factors that still need to be defined. Our study makes an important contribution to this bi-functional nature of PLK1.

and lines 417-426:

Since two distinct EBNA2 mutants that both target independent PLK1 related functions of the EBNA2/PLK1 complex promote cancerogenesis, we conclude that PLK1 might act as a tumor suppressor in EBNA2 driven lymphomagenesis. Until to date, the interaction of EBNA2 with PLK1 was unknown and thus latent EBV was not considered a risk factor in PLK1 inhibitor

based therapeutic strategies. Knowing that latent EBV is ubiquitously found in humans, PLK1 inhibitors could cause adverse effects with detrimental consequences for immune-deficient patients, which are prone to EBV related cancerogenesis. Humanized mice infected with EBV and specific EBV mutants will serve as pre-clinical animal models to study the action of PLK1 inhibitors in the context of latent EBV infection.

Referee #2

This is a nice complete study identifying a new way that host cells can restrict the activity of the key Epstein-Barr virus transcription factor EBNA2. EBNA2 is an essential driver of the B cell transformation that enables EBV persistence in B cells and can ultimately lead to tumour development. Increasing evidence indicates that host cells use multiple mechanisms to suppress EBNA2 activity and that sequence variation in EBNA2 can affect its transactivation activity. This manuscript describes the identification of a new EBNA2 binding partner by mass spectrometry, the mitotic kinase PLK1, the mapping of its docking site (primed by phosphorylation by CDK1) and its phosphotarget sites in EBNA2. The authors demonstrate that PLK1 phosphorylation impairs EBNA2 transactivation activity in reporter assays and that recombinant viruses with mutations in PLK1 docking or phosphorylation sites drive the increased proliferation of infected B cells. These mutant viruses also establish B cell infections in humanized mice more rapidly and show increased tumour promoting properties linking the biochemical characterisation to an in vivo phenotype. Experiments are well executed and controlled, and conclusions are sound.

We thank referee 2 for the positive comments on our manuscript and suggestions for improvement.

Suggestions for improvements are relatively minor:

1. Figure 3. S184A phosphorylation does seem to be reduced, contrary to the text, although transactivation assays rule out a functional role. Some additional phosphorylation sites may be mapped with lower confidence scores, rather than ambiguously.

We agree that we should mention the weak phosphorylation of mutant S184A in the text and have modified the text in lines 179-181:

Of these, five phosphorylation sites (S184, S258, S457, S479 and T465) were confidently localized (Fig. EV2) and 6 additional sites (T175, T178, T263, T267, T464 and S266) were mapped ambiguously with less confidence.

We also revised the text in lines 186-189:

In the absence of Volasertib, phosphorylation of S184A was slightly reduced, while phosphorylation of S258A and S479A was not reduced. Phosphorylation of the EBNA2 mutants S457A and T465V was severely impaired, and the combination of both mutations (S457A/T465V) abolished phosphorylation (Fig. 3E).

2. It would be good to identify LMP1 and MYC as known EBNA2 target genes for the non EBV expert.

In response to the referee we revised the text of the manuscript to explain the specific functions of MYC and LMP1 in the context of EBV B cell immortalization in more detail.

The following paragraph has been added to the introduction on page 2/3 lines 69-79:

EBNA2 can activate the transcription of the viral latent membrane protein 1 (LMP1) and the cellular MYC gene in the absence of de novo protein synthesis. Thus, both are direct target genes of EBNA2 (Kaiser *et al*, 1999). Both proteins can induce an avalanche of secondary EBNA2 target genes. LMP1 promotes proliferation and cell survival. This multifunctional membrane oncoprotein activates the cellular NFκB, JNK, and STAT signaling cues and thereby mimics CD40 signaling (Thorley-Lawson, 2001). The proto-oncoprotein and transcription factor MYC stimulates global RNA and protein synthesis to foster cell growth of quiescent lymphocytes and cell cycle progression in normal non-malignant cells. High-level MYC expression is associated with lymphomagenesis but in the absence of co-stimulatory signals it may also induce apoptosis (Baluapuri *et al*, 2020).

The difference in impact on LMP1 activation by the mutants compared to MYC could be discussed. Could this relate to differences in dependency on p300 for activation?

Lines 335-340 of this manuscript discuss the potential impact of p300 binding on EBNA2 S457A/T465V activity. Since p300 is not the only transcriptional co-factor that binds to the EBNA2 TAD it would be very difficult to define the exact mechanism that causes the differential response of the MYC and the LMP1 gene to EBNA2 S457A/T465V. Several candidate proteins would have to be considered.

EBNA2 from type 2 EBV strains shows reduced activation of a subset of target genes including LMP1. Have the authors looked at gene activation profiles more widely? Can they draw any parallels?

Both residues, S457 and T465, are conserved in EBV type1 and type 2 genomes. We have not performed any genome wide transcriptome analyses, but anticipate that the results would be dominated by the impact of the elevated LMP1 levels on the cellular gene expression. Further epigenetic studies would be required to define EBNA2 co-binding factors in the context of LMP1 and MYC enhancers. These experiments would be very interesting but are beyond the scope of this project.

3. I am not sure that I agree that 'EBNA2 activity needs to be controlled by PLK1'. Perhaps this is better described as a mechanism that is mutually beneficial to host and virus- reducing transformation and allowing establishment of latency.

We agree with referee 2 and have changed lines 372 -378:

PLK1 restrains the growth transformation potential of EBNA2 to establish tumor free survival of latently infected hosts.
Both mutants independently target highly conserved amino acid residues (New Figure EV3) but surprisingly both are gain of function mutants, which exhibit improved transformation capacities. We conclude that the control of EBNA2 activity is evolutionary conserved and might be

beneficial for host and virus to protect against cancerogenesis and establish and stabilize long-term latency and thus the viral reservoir in the host.

4. The discussion section is quite repetitive of the results, often restating them. Could this section be used to expand on context and further hypotheses more? For example, could the balance of PLK1 phosphorylation and dephosphorylation be important in controlling transcription in interphase. Are these phosphorylation sites conserved across EBV strains?

In response to this comment of referee 2 we have revised several passages of the Discussion section. We also included a new Figure EV3, which demonstrates the conservation of the respective residues.

5. Clarity of writing and use of English could be improved in places and there are incomplete/incorrect Figure citations (e.g. p4). Figure 1 legend and details do not state the identity of the cell lines used. The order of Figure 1 panels is not logical. The text on p8 implies that T cells are infected with mutant viruses.

We changed the text in line 278.

We have carefully checked for mistakes and incorrect citations. The identity of the cells in Figures 1 and 2 has been clarified. The panels in Figure 2 have been restructured.

Referee #3

This manuscript provides new and exciting data on how EBNA2 is regulated and promotes oncogenesis. The study is initiated with a simple IP/MS experiment of tagged EBNA2 in DG75 cells. This brings down known interactors among 19 proteins and includes PLK1 as a novel interactor. The interaction is validated by western blotting and mutagenesis experiments suggests the PLK1 binding region of EBNA2 is in the C-terminus. Alignment of EBNA2 peptides with known PLK1 substrates identifies three potential sites where a CDK1 priming phosphorylation may enable PLK1 binding. Mutagenesis narrows this down to S379 and convincing evidence is provided for in vitro kinase (CDK1) activity and specific PLK1 binding of the phospho peptide as well as IP of EBNA2/PLK1 in cells. Next, EBNA2 is found to increase PLK1 activity and mutation of two key residues (S457 and T465) is found to ablate EBNA2 phosphorylation by PLK1. Interestingly, mutation of these residues increases EBNA2 transactivation potential on a CBF1-multimerized promoter. Furthermore, this mutant has increased association with the co-activator, p300 supporting the idea that PLK1 phosphorylation suppresses EBNA2 transcriptional activity, possibly through restricting p300 binding.

WT PLK1, but not a kinase dead mutant, suppressed EBNA2 activity when over-expressed, but this was lost on the PLK1 binding mutant, which robustly transactivated. Interestingly, the CDK1 priming mutant (S379A) was inhibited by PLK1 suggesting that the priming phosphorylation isn't required (at least in over-expression conditions) for PLK1 inhibition of EBNA2 activity.

Mutant EBV genomes were generated with S379A or S457A/T465V and interestingly these mutants both promoted outgrowth of latently infected B cells more so than wt virus. Carrying this phenotype over to huNSG mice, the authors find that the double mutant (which is unable to be suppressed by PLK1) kills mice, while the S379A and wt do not in this model of infection

following CD34+ cell-mediated immune reconstitution. The difference in tumor burden is subtle, but notable where there is a higher proportion of macroscopically visible tumors in the double mutant mice (and perhaps single mutant). However, EBV viral loads in the spleen and blood are largely similar, though the mutant infections trend higher in the spleen and display a significant boost at 3 weeks post infection (the double mutant). Overall, these data paint an interesting molecular picture of EBNA2 regulation that follows on some earlier work by the Pagano lab indicating that EBNA2 transcriptional activity was suppressed by mitotic kinases.

Specific comments:
1. It would be interesting to know what the conservation of these aa residues are among EBV strains (eg Farrell data set), type 1/2, and even back through NHPs.

We thank referee 3 for this suggestion and have added the alignment of several EBV strains type 1 and 2 as well as the related lymphocryptoviruses of the non-human primates Baboon and Macaque as a new Figure EV3 to the manuscript. The results of Figure EV3 are described in line 180-185:

The alignment (Figure EV3) of the protein sequence of different EBV type 1 or type 2 strains or related non-human primate lymphocryptoviruses (LCV) revealed that residue S184 and S258 were conserved in type 1 EBV strains, S379 and S479 were identical across species while S457 was conserved across human sequences. T465 was identical across human sequences and replaced by the similar S residue in LCV.

and discussed in lines 340-344:

The alignment of EBV and LCV protein sequences revealed a high conservation of all three residues across EBV strains. S379 and T465 were conserved even across species. Considering that the respective mutants are gain of function i.e. activated proteins, we think that PLK1 contributes to the establishment of latency by attenuating EBNA2 activity.

and discussed in lines 375-379:

Both mutants independently target highly conserved amino acid residues (Figure EV3) but surprisingly both are gain of function mutants, which exhibit improved transformation capacities. We conclude that the control of EBNA2 activity is evolutionary conserved and might be beneficial for host and virus to protect against cancerogenesis and establish and stabilize long-term latency and thus the viral reservoir in the host.

2. It appears that the double mutant may be expressed lower in LCLs, could there be an impact on turnover (which negatively correlates with transcriptional activity?)

Indeed, in particular protein levels of EBNA2 S457A/T465V of established LCLs are consistently lower than EBNA2 wt levels as mentioned in lines 244-247. In contrast, transient expression of the double mutant did not result in significantly lower expression levels. Thus, we do not assume that the double mutant is specifically prone to high turnover rates. We rather speculate that high-level expression of this very active mutant could be incompatible with long-term proliferation of the infected cells and the cells expressing less protein have been selected over time. Since we cannot provide any evidence that supports this idea, we do not want to discuss this hypothesis in the manuscript in more detail.

3. A transformation assay may provide better resolution on long-term impact of the mutants versus the short-term proliferation assay.

We thank referee 3 that (s)he brought up this point but we are convinced that the combined analysis of short-term proliferation assays early after infection and of established cell lines is more informative than transformation assays. The reasons are the following: For a transformation assay, primary B cells would have been seeded in micro-wells and the cultures are infected with the respective viral preparations. The tests are performed under limiting dilution conditions using either different numbers of B cells per culture or different amounts of each virus. After 3-6 weeks the fraction of proliferating cultures is determined. Since in our case all infected cultures proliferate, although at different rates, we expect that cultures with a high potential to proliferate would grow to maximal density, starve and loose viability while slow growing cells can survive longer. Unfortunately, we do not have a device that can monitor the proliferation of suspension cell cultures in real-time. Thus, between seeding/infection and the final read-out, there is a black-box, and we do not expect an improved resolution on long-term impact. We thus decided to analyze the early and a late phase of the transformation process. We have performed short term proliferation assays on freshly infected primary B cells (Figure 5A) and have counted cell numbers of already long-term established LCLs (Figure 5B) for a time period of 6 days. In essence, we obtained very similar results in both settings.

4. Given the modest phenotype on tumorigenesis, perhaps the title should be adjusted to indicate that PLK1 restrains EBNA2 activity.

The experimental regimen that we followed was restricted to a fixed time window in order to comply with the rules of animal welfare. All infected animals were sacrificed after 5 weeks also in case they had not developed any symptoms or earlier in case of weight loss of more than 20%. Based on this limited time frame we can only estimate the severity of the phenotype but consider it rather pronounced than modest, since, at later time points, mortality rates as well as tumor incidences and clonality might have been more pronounced.

We thus have modified the title of our manuscript to:

PLK1-dependent phosphorylation restrains EBNA2 activity and lymphomagenesis in EBV-infected mice.

1st Revision - Editorial Decision 9th Jul 2021

Dear Dr. Kempkes,

Thank you for the submission of your revised manuscript to our editorial offices. We have now received the reports from the three referees that were asked to re-evaluate your study, you will find below. As you will see, the referees now fully support the publication of your study.

Before we can proceed with formal acceptance, I have these editorial requests I ask you to address in a final revised manuscript:

- Please order the manuscript sections (using this nomenclature) like this: Title page - Abstract - Introduction - Results - Discussion - Materials and Methods - DAS (data availability section) - Acknowledgements - Author contributions - Conflict of interest - References - Figure legends - Expanded View Figure legends - Table with legend.

- The title is presently minimally too long (it should have not more than 100 characters including spaces). Could it be shortened to:
PLK1-dependent phosphorylation restrains EBNA2 activity and lymphomagenesis in EBV-infected mice

- Please move Table S1 and Table EV1 to an Appendix file. Please change their names to "Appendix Table S1" and "Appendix Table S2" and change their callouts accordingly. The Appendix file should be a pdf file with page numbers and a table of contents (TOC) on the first page including page numbers. Please add all legends and further information below the two tables in the Appendix file, and finally upload this as Appendix. Finally, please remove any legends/descriptions of the two tables from the main manuscript text.

- Fig. EV1 looks very blurred and out of focus. Please provide a figure file with better resolution.

- For Figure EV4A, please add a black line or a white space clearly indicating that the image was put together from two different gels.

- Please name the Data Availability Section just like this (remove the word Resources). Please also remove now the referee access and make sure that the dataset is public upon publication of the paper.

- Please make sure that all the funding information is entered also into the online submission system and is complete and similar to the one in the manuscript text file.

- Regarding data quantification and statistics, please make sure that the number "n" for how many independent experiments were performed, their nature (biological versus technical replicates), the bars and error bars (e.g. SEM, SD) and the test used to calculate p-values is indicated in the respective figure legends (also of the EV). Please provide error bars and statistical testing where applicable.

- As the Western blots shown are significantly cropped, please provide the source data for all the blots (main and EV figures). The source data will be published in a separate source data file online along with the accepted manuscript and will be linked to the relevant figures. Please submit the source data (scans of entire blots) together with the final revised manuscript. Please include size

markers for scans of entire blots, label the scans with figure and panel number and send one PDF file per figure (main and EV figures).

- Finally, please find attached a word file of the manuscript text (provided by our publisher) with changes we ask you to include in your final manuscript text, and some queries, we ask you to address. Please provide your final manuscript file with track changes, in order that we can see any modifications done.

In addition, I would need from you:
- a short, two-sentence summary of the manuscript (35 words).
- three to four bullet points highlighting the key findings of your study.
- a schematic summary figure (synopsis image) in jpeg or tiff format with the exact width of 550 pixels and a height of not more than 400 pixels that can be used as a visual synopsis on our website.

I look forward to seeing the final revised version of your manuscript when it is ready. Please let me know if you have questions regarding the revision.

Kind regards,

Achim Breiling
Editor
EMBO Reports

Referee #1:

I have reviewed all the changes by the authors and they have satisfactory addressed all the major concerns of the reviewers. This is solid work and demonstrates a role for PLK1 in EBV associated cancers.

Referee #2:

All points have been addressed or responded to satisfactorily.

Referee #3:

The authors have adequately responded to all of my concerns.

The authors have addressed all minor editorial requests.

2nd Revision - Editorial Decision 10th Sep 2021

Bettina Kempkes
Helmholtz-muenchen.de
Department of Gene Vectors
Feodor-Lynenstr. 21
Germany

Dear Bettina,

Thank you for implementing the last changes required. I am now very pleased to accept your manuscript for publication in the next available issue of EMBO reports. Thank you for your contribution to our journal.

At the end of this email I include important information about how to proceed. Please ensure that you take the time to read the information and complete and return the necessary forms to allow us to publish your manuscript as quickly as possible.

As part of the EMBO publication's Transparent Editorial Process, EMBO reports publishes online a Review Process File to accompany accepted manuscripts. As you are aware, this File will be published in conjunction with your paper and will include the referee reports, your point-by-point response and all pertinent correspondence relating to the manuscript.

If you do NOT want this File to be published, please inform the editorial office within 2 days, if you have not done so already, otherwise the File will be published by default [contact: emboreports@embo.org]. If you do opt out, the Review Process File link will point to the following statement: "No Review Process File is available with this article, as the authors have chosen not to make the review process public in this case."

Should you be planning a Press Release on your article, please get in contact with emboreports@wiley.com as early as possible, in order to coordinate publication and release dates.

Please note that under the DEAL agreement of German scientific institutions with our publisher Wiley, your paper might be eligible for open access publication in a way that is free of charge for the authors. Please contact either the administration at your institution or our publishers at Wiley (emboreports@wiley.com) for further questions.
(see also https://authorservices.wiley.com/author-resources/Journal-Authors/open-access/affiliation-policies-payments/institutional-funder-payments.html

Thank you again for your contribution to EMBO reports and congratulations on a successful publication. Please consider us again in the future for your most exciting work.

Kind regards,

Martina

Martina Rembold, PhD
Senior Editor

EMBO reports

****

THINGS TO DO NOW:

You will receive proofs by e-mail approximately 2-3 weeks after all relevant files have been sent to our Production Office; you should return your corrections within 2 days of receiving the proofs.

Please inform us if there is likely to be any difficulty in reaching you at the above address at that time. Failure to meet our deadlines may result in a delay of publication, or publication without your corrections.

All further communications concerning your paper should quote reference number EMBOR-2021-53007V3 and be addressed to emboreports@wiley.com.

Should you be planning a Press Release on your article, please get in contact with emboreports@wiley.com as early as possible, in order to coordinate publication and release dates.

EMBO PRESS

YOU MUST COMPLETE ALL CELLS WITH A PINK BACKGROUND ➔

PLEASE NOTE THAT THIS CHECKLIST WILL BE PUBLISHED ALONGSIDE YOUR PAPER

Corresponding Author Name: Bettina Kempkes

Journal Submitted to: EMBO Reports

Manuscript Number: EMBOR-2021-53007V3

Reporting Checklist For Life Sciences Articles (Rev. June 2017)

This checklist is used to ensure good reporting standards and to improve the reproducibility of published results. These guidelines are consistent with the Principles and Guidelines for Reporting Preclinical Research issued by the NIH in 2014. Please follow the journal's authorship guidelines in preparing your manuscript.

A- Figures

1. Data

The data shown in figures should satisfy the following conditions:

- the data were obtained and processed according to the field's best practice and are presented to reflect the results of the experiments in an accurate and unbiased manner.
- figure panels include only data points, measurements or observations that can be compared to each other in a scientifically meaningful way.
- graphs include clearly labeled error bars for independent experiments and sample sizes. Unless justified, error bars should not be shown for technical replicates.
- if n< 5, the individual data points from each experiment should be plotted and any statistical test employed should be justified
- Source Data should be included to report the data underlying graphs. Please follow the guidelines set out in the author ship guidelines on Data Presentation.

2. Captions

Each figure caption should contain the following information, for each panel where they are relevant:

- a specification of the experimental system investigated (eg cell line, species name).
- the assay(s) and method(s) used to carry out the reported observations and measurements
- an explicit mention of the biological and chemical entity(ies) that are being measured.
- an explicit mention of the biological and chemical entity(ies) that are altered/varied/perturbed in a controlled manner.

- the exact sample size (n) for each experimental group/condition, given as a number, not a range;
- a description of the sample collection allowing the reader to understand whether the samples represent technical or biological replicates (including how many animals, litters, cultures, etc.).
- a statement of how many times the experiment shown was independently replicated in the laboratory.
- definitions of statistical methods and measures:
  - common tests, such as t-test (please specify whether paired vs. unpaired), simple χ^2 tests, Wilcoxon and Mann-Whitney tests, can be unambiguously identified by name only, but more complex techniques should be described in the methods section;
  - are tests one-sided or two-sided?
  - are there adjustments for multiple comparisons?
  - exact statistical test results, e.g., P values = x but not P values < x;
  - definition of 'center values' as median or average;
  - definition of error bars as s.d. or s.e.m.

Any descriptions too long for the figure legend should be included in the methods section and/or with the source data.

In the pink boxes below, please ensure that the answers to the following questions are reported in the manuscript itself. Every question should be answered. If the question is not relevant to your research, please write NA (non applicable). We encourage you to include a specific subsection in the methods section for statistics, reagents, animal models and human subjects.

USEFUL LINKS FOR COMPLETING THIS FORM

http://www.antibodypedia.com
http://1degreebio.org
http://www.equator-network.org/reporting-guidelines/improving-bioscience-research-repor

http://grants.nih.gov/grants/olaw/olaw.htm
http://www.mrc.ac.uk/Ourresearch/Ethicsresearchguidance/Useofanimals/index.htm
http://ClinicalTrials.gov
http://www.consort-statement.org
http://www.consort-statement.org/checklists/view/32-consort/66-title
http://www.equator-network.org/reporting-guidelines/reporting-recommendations-for-tumo
http://datadryad.org
http://figshare.com
http://www.ncbi.nlm.nih.gov/gap
http://www.ebi.ac.uk/ega
http://biomodels.net/
http://biomodels.net/miriam/
http://jjj.biochem.sun.ac.za
http://oba.od.nih.gov/biosecurity/biosecurity_documents.html
http://www.selectagents.gov/

B- Statistics and general methods

Please fill out these boxes ➔ (Do not worry if you cannot see all your text once you press return)

1.a. How was the sample size chosen to ensure adequate power to detect a pre-specified effect size?	All cell culture experiments were performed in technical triplicates and 3 biological replicates
1.b. For animal studies, include a statement about sample size estimate even if no statistical methods were used.	For animal studies the number of animals per biological replicate was n=5.
2. Describe inclusion/exclusion criteria if samples or animals were excluded from the analysis. Were the criteria pre-established?	In case animals had to be sacrificed or died prior to the planned date (5 weeks post-infection) this is specifically mentioned in the text.
3. Were any steps taken to minimize the effects of subjective bias when allocating animals/samples to treatment (e.g. randomization procedure)? If yes, please describe.	NA
For animal studies, include a statement about randomization even if no randomization was used.	The animals were ascribed to a distinct experimental group ensuring similar ratios of males to females and similar reconstitution levels and immune cell activation in the peripheral blood.
4.a. Were any steps taken to minimize the effects of subjective bias during group allocation or/and when assessing results (e.g. blinding of the investigator)? If yes please describe.	No blinding was done
4.b. For animal studies, include a statement about blinding even if no blinding was done	No blinding was done
5. For every figure, are statistical tests justified as appropriate?	The statistical tests are specified in the figure legends.
Do the data meet the assumptions of the tests (e.g., normal distribution)? Describe any methods used to assess it.	NA
Is there an estimate of variation within each group of data?	NA

Is the variance similar between the groups that are being statistically compared?	NA

C- Reagents

6. To show that antibodies were profiled for use in the system under study (assay and species), provide a citation, catalog number and/or clone number, supplementary information or reference to an antibody validation profile. e.g., Antibodypedia (see link list at top right), 1DegreeBio (see link list at top right).	Antibody sources are listed in the Material and Methods section.
7. Identify the source of cell lines and report if they were recently authenticated (e.g., by STR profiling) and tested for mycoplasma contamination.	DG75 cells were obtained from Leibniz Institute DSMZ-German Collection of Microorganisms and Cell Cultures (ACC 83).

* for all hyperlinks, please see the table at the top right of the document

D- Animal Models

8. Report species, strain, gender, age of animals and genetic modification status where applicable. Please detail housing and husbandry conditions and the source of animals.	All details have been specified in the Material and Metods section.
9. For experiments involving live vertebrates, include a statement of compliance with ethical regulations and identify the committee(s) approving the experiments.	The respective animal protocol (ZH159-17/ZH008-20) was approved by the veterinary office of the canton of Zurich, Switzerland
10. We recommend consulting the ARRIVE guidelines (see link list at top right) (PLoS Biol. 8(6), e1000412, 2010) to ensure that other relevant aspects of animal studies are adequately reported. See author guidelines, under 'Reporting Guidelines'. See also: NIH (see link list at top right) and MRC (see link list at top right) recommendations. Please confirm compliance.	We assure compliance.

E- Human Subjects

11. Identify the committee(s) approving the study protocol.	NA
12. Include a statement confirming that informed consent was obtained from all subjects and that the experiments conformed to the principles set out in the WMA Declaration of Helsinki and the Department of Health and Human Services Belmont Report.	NA
13. For publication of patient photos, include a statement confirming that consent to publish was obtained.	NA
14. Report any restrictions on the availability (and/or on the use) of human data or samples.	NA
15. Report the clinical trial registration number (at ClinicalTrials.gov or equivalent), where applicable.	NA
16. For phase II and III randomized controlled trials, please refer to the CONSORT flow diagram (see link list at top right) and submit the CONSORT checklist (see link list at top right) with your submission. See author guidelines, under 'Reporting Guidelines'. Please confirm you have submitted this list.	NA
17. For tumor marker prognostic studies, we recommend that you follow the REMARK reporting guidelines (see link list at top right). See author guidelines, under 'Reporting Guidelines'. Please confirm you have followed these guidelines.	NA

F- Data Accessibility

18: Provide a "Data Availability" section at the end of the Materials & Methods, listing the accession codes for data generated in this study and deposited in a public database (e.g. RNA-Seq data: Gene Expression Omnibus GSE39462, Proteomics data: PRIDE PXD000208 etc.) Please refer to our author guidelines for 'Data Deposition'.<br><br>Data deposition in a public repository is mandatory for:<br>a. Protein, DNA and RNA sequences<br>b. Macromolecular structures<br>c. Crystallographic data for small molecules<br>d. Functional genomics data<br>e. Proteomics and molecular interactions	The mass spectrometry proteomics data have been deposited in the ProteomeXchange Consortium via the PRIDE partner repository with the dataset identifier PXD022970.
19. Deposition is strongly recommended for any datasets that are central and integral to the study; please consider the journal's data policy. If no structured public repository exists for a given data type, we encourage the provision of datasets in the manuscript as a Supplementary Document (see author guidelines under 'Expanded View' or in unstructured repositories such as Dryad (see link list at top right) or Figshare (see link list at top right).	N/A
20. Access to human clinical and genomic datasets should be provided with as few restrictions as possible while respecting ethical obligations to the patients and relevant medical and legal issues. If practically possible and compatible with the individual consent agreement used in the study, such data should be deposited in one of the major public access-controlled repositories such as dbGAP (see link list at top right) or EGA (see link list at top right).	N/A
21. Computational models that are central and integral to a study should be shared without restrictions and provided in a machine-readable form. The relevant accession numbers or links should be provided. When possible, standardized format (SBML, CellML) should be used instead of scripts (e.g. MATLAB). Authors are strongly encouraged to follow the MIRIAM guidelines (see link list at top right) and deposit their model in a public database such as Biomodels (see link list at top right) or JWS Online (see link list at top right). If computer source code is provided with the paper, it should be deposited in a public repository or included in supplementary information.	N/A

G- Dual use research of concern

22. Could your study fall under dual use research restrictions? Please check biosecurity documents (see link list at top right) and list of select agents and toxins (APHIS/CDC) (see link list at top right). According to our biosecurity guidelines, provide a statement only if it could.	No
